# Immunologic Changes during Pandemic (H1N1) 2009, China

**DOI:** 10.3201/eid1706.100643

**Published:** 2011-06

**Authors:** Hong-Hui Shen, Jun Hou, Wei-Wei Chen, Bing-Ke Bai, Hai-Bin Wang, Tong-Sheng Guo, Ai-Xia Liu, Yong-Li Li, Min Zhao, Pan-Yong Mao, Jin Li, Bo-An Li, Yuan-Li Mao

**Affiliations:** Author affiliation: Beijing 302 Hospital, Beijing, People’s Republic of China

**Keywords:** influenza, pandemic (H1N1) 2009 virus, viruses, immunologic changes, lymphocyte subpopulations, cytokines, antibody production, China, dispatch

## Abstract

We analyzed changes in immunologic values over time for 28 hospitalized patients with pandemic (H1N1) 2009. Levels of interleukin-6, interferon-γ, and interleukin-10 increased 1 day after illness onset and then decreased to baseline levels. Levels of virus-specific antibody were undetectable 1 day after illness onset and peaked 36 days later.

Pandemic (H1N1) 2009 virus emerged in March 2009 and spread worldwide (*1**,**2*). Most patient laboratory data result from samples or information obtained on the day of hospitalization or when the patient was experiencing the acute phase of infection. Changes in laboratory data throughout the disease course have rarely been reported. We retrospectively analyzed immunologic changes for hospitalized patients through the entire course of pandemic (H1N1) 2009. These data may provide a better understanding of disease pathogenesis.

## The Study

Twenty-eight patients admitted to Beijing 302 Hospital in Beijing, China, were enrolled in this study in September 2009. All patients had mild clinical courses and fulfilled the case definition for pandemic (H1N1) 2009 (*3*). Median interval from onset of illness to hospitalization was 1 day (range 0–3 days), and median time of hospitalization was 7 days (range 6–14 days). Serum samples were obtained from patients 1 day after illness onset and 4, 14, 36, and 48 days later for cytokine and antibody measurement. Serum samples from 22 healthy persons were used to determine cytokine and antibody baseline levels.

Serum cytokine concentrations were detected by using the BD Cytometric Bead Array Human Cytokine Kit (BD Biosciences, San Jose, CA, USA) and a BD FACSCalibur flow cytometer, according to the manufacturer’s instructions. CD3 T-lymphocyte counts were <690 cells/mm^3^ in 9 (32.1%) of 28 patients and represented <55% of total lymphocyte counts in 4 (14.3%) patients (Table). CD4 T-lymphocyte counts were <400 cells/mm^3^ in 13 (46.4%) patients and represented <31% of total lymphocyte counts in 8 (28.6%) patients. CD8 T-lymphocyte counts were <190 cells/mm^3^ in 3 (10.7%) patients. B-cell counts were <90 cells/mm^3^ in 1 (3.6%) patient. Natural killer cells represented >27% of lymphocyte counts in 4 (14.3%) patients. Fourteen (50.0%) patients had a CD4:CD8 ratio less than the standard reference ratio of 1.4.

**Table:** Flow cytometric analysis of peripheral blood lymphocyte subsets for 28 patients with pandemic (H1N1) 2009 at hospitalization, China*

Lymphocyte subset	No. positive/no. tested (%)
CD45 <1,500 cells/mm^3^†	21/28 (75.0)
CD3	
<690 cells/mm^3^	9/28 (32.1)
<55%	4/28 (14.3)
CD4	
<400 cells/mm^3^	13/28 (46.4)
<31%	8/28 (28.6)
CD8	
<190 cells/mm^3^	3/28 (10.7)
<13%	0/28 (0)
B cells	
<90 cells/mm^3^	1/28 (3.6)
<6%	0/28 (0)
Natural killer cells	
>590 cells/mm^3^	1/28 (3.6)
>27%	4/28 (14.3)
Ratio of CD4:CD8 cells <1.4	14/28 (50.0)

*Lymphocyte subsets were defined as the following combinations: CD3+/CD45+ for CD3 T-lymphocytes; CD3+/CD4/+/CD45+ for CD4 T-lymphocytes; CD3+/CD8+/CD45+ for CD8 T-lymphocytes; CD19+/CD45+ for B-lymphocytes; and CD3+/CD(16+56)+/CD45+ for natural killer cells. †CD45 lymphocyte count was designated the total lymphocyte count.

Flow cytometric results showing development of peripheral blood lymphocyte subsets during the disease course were divided into 3 groups on the basis of time of illness onset until date of blood sample collection: 1–3, 4–6, or 7–10 days. These groups were used because the longest time from onset of illness to hospitalization was 3 days for all patients, and the peak temperature for patients was observed 3 days after illness onset.

**Figure 1:**
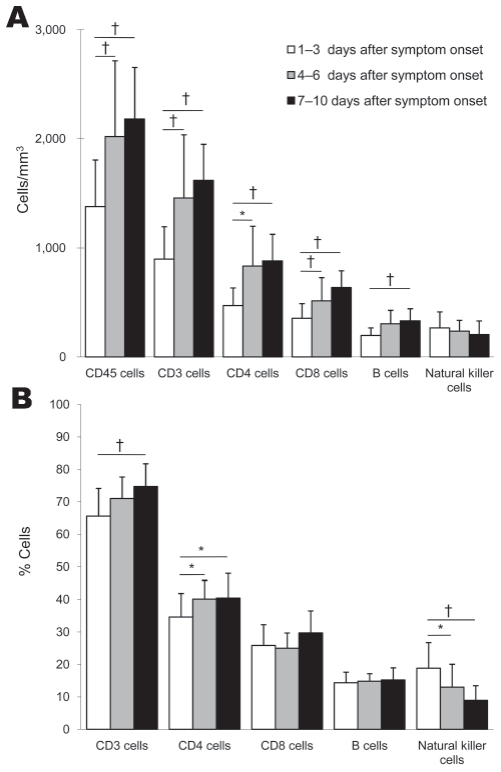
Flow cytometric analysis of peripheral blood lymphocyte subset counts of 28 patients with pandemic (H1N1) 2009, China. Counts and percentages are means. Error bars indicate SD. Each mean value was within the corresponding reference range. Lymphocyte subsets are as shown in the Table. A) Absolute count. B) Percentage of lymphocyte subset count compared with total lymphocyte count. *p<0.05; †p<0.01.

Mean counts and percentages of all T- and B-lymphocyte subsets increased after 3 days of illness compared with results obtained during the first 3 days of illness (Figure 1). However, the increase in CD8 and B cells was not significant. Another study showed a decrease in CD4, CD8, and B cells ≤2 days of symptom onset in patients with pandemic (H1N1) 2009 than in healthy persons (*4*). Our results show impaired adaptive immune responses and a gradual increase during recovery in mildly affected patients.

**Figure 2:**
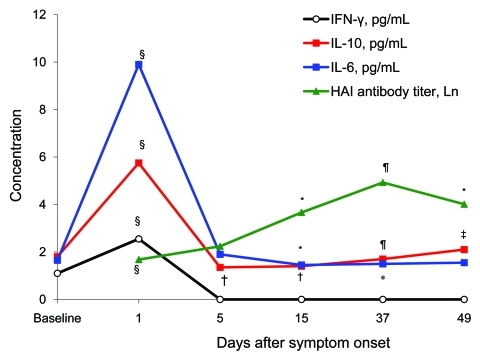
Serum cytokine concentrations and hemagglutination inhibition (HAI) antibody titers of 28 patients with pandemic (H1N1) 2009 during hospitalization and the follow-up period 15, 37, and 49 days after symptom onset, China. Serum concentrations of interferon-γ (IFN-γ), interleukin-10 (IL-10), and IL-6 are medians (pg/mL). Serum HAI antibody titers were transformed by using the natural logarithm and are shown as means. Baseline cytokine concentrations on the y-axis are values for healthy persons. *p<0.05 when IL-6 or HAI antibody levels were compared with those at day 5; †p<0.05 when IL-10 level was compared with those at baseline; ‡p<0.05 when IL-10 level was compared with those at days 5 or 15; §p<0.05 when value was compared with that at any other time point; ¶p<0.05 when value was compared with those at days 5, 15, or 49.

We measured serum cytokine concentrations and hemagglutination inhibition (HAI) antibody titers in patients during hospitalization and the follow-up period (Figure 2). We observed an increase in interleulin-6 (IL-6) levels 1 day after illness onset, which were 6.0-fold higher than the baseline level, and a 2.3-fold increase in interferon-γ (IFN-γ) levels. These levels decreased to baseline levels 5 days after illness onset, although the IL-6 level 5 days after illness onset was higher than levels 15 and 37 days after illness onset. The maximum IL-10 level 1 day after symptom onset was 3.2-fold higher than the baseline level. This level decreased to a value lower than the baseline level within 4 days, and then gradually increased to the baseline level 37 days after illness onset. Serum IL-6, IFN-γ, and IL-10 levels were not related to patient temperature 1 day after symptom onset, peak temperature during the disease, or period of fever. These levels showed minor differences that were not related to cough or sore throat in patients.

Only 1 patient had an HAI antibody titer ≥10 (titer 20) 1 day after illness onset. The HAI geometric mean titer increased 5 days after symptom onset compared with that 1 day after symptom onset and continued to increase until it reached a peak level of 137.9 at 37 days after symptom onset (25.5-fold increase). Peak HAI antibody titers ≥40 and ≥4-fold increases were observed in 27 (96.4%) patients.

## Conclusions

Bermejo-Martin et al. reported increased serum levels of IL-6, IFN-γ, and tumor necrosis factor-α in patients with pandemic (H1N1) 2009 during the first 5 days after symptom onset; no difference in levels of these 3 cytokines was observed in patients with mild disease and controls (*5*). However, similar to another report (*4*), we detected increases of IL-6 and IFN-γ levels in patients with mild disease during the first 3 days after symptom onset. These different patterns may be caused by different intervals from time of symptom onset to date of sample collection (5 days vs. 3 days) because IL-6 and IFN-γ levels in our study quickly decreased to baseline levels ≤7 days after symptom onset. These results suggest that serum IL-6 and IFN-γ levels may be increased in patients with pandemic (H1N1) 2009 within the first 3 days after symptom onset, followed by a decrease to baseline levels ≤5 days after symptom onset in patients with mild disease or a continuous increase in severely affected patients.

IL-6 and IFN-γ are associated with antiviral immune responses during influenza infection (*6**–**8*). However, continuous, excessive release of IL-6 three days after illness onset likely contributed to serious pulmonary inflammation and tissue injury, as has been documented for severe acute respiratory syndrome and 1918 pandemic influenza, but this release could be tempered by production of IL-10 (*6**,**7**,**9**–**11*).

The proportion of persons 18–60 years of age with a ≥4-fold increase in HAI titer who received 1 dose (15 μg) of monovalent pandemic (H1N1) 2009 nonadjuvant vaccine was 96.2%, and the proportion with an increased HAI titer ≥40 was 97.1%, results similar to those of a recent study (*12*). However, the geometric mean titer in healthy vaccinated persons was 237.8, a 34.5-fold increase over the prevaccination titer, which was greater than that for patients naturally infected with pandemic (H1N1) 2009 virus (*12*). This finding may have resulted from impaired adaptive immune responses against pandemic (H1N1) 2009 virus in the initial phase, which included decreased numbers of CD4 and B lymphocytes and an increase in T regulatory cells (*4*).

In conclusion, our data indicated changes in cellular profiles during pandemic (H1N1) 2009 virus infection; showed that transient production of IL-6, IFN-γ, and IL-10 are main effectors of the early innate immune response against pandemic (H1N1) 2009 virus; and indicated that adaptive immune responses are impaired in the initial phase after infection. These factors may help clarify the pathogenesis of pandemic (H1N1) 2009 virus and provide new approaches in overcoming severe infections.
